# PLGA-PEG-ANG-2 Nanoparticles for Blood–Brain Barrier Crossing: Proof-of-Concept Study

**DOI:** 10.3390/pharmaceutics12010072

**Published:** 2020-01-17

**Authors:** Gina P. Hoyos-Ceballos, Barbara Ruozi, Ilaria Ottonelli, Federica Da Ros, Maria Angela Vandelli, Flavio Forni, Eleonora Daini, Antonietta Vilella, Michele Zoli, Giovanni Tosi, Jason T. Duskey, Betty L. López-Osorio

**Affiliations:** 1 Grupo de Investigación Ciencia de los Materiales, Instituto de Química, Facultad de Ciencias Exactas y Naturales, Universidad de Antioquia, Calle 62 No. 52–59, Medellín 050015, Colombia; ginapao711@gmail.com; 2 Department of Life Sciences, University of Modena and Reggio Emilia, 41124 Modena, Italy; barbara.ruozi@unimore.it (B.R.); ilaria.ottonelli@unimore.it (I.O.); federica.daros93@gmail.com (F.D.R.); vandelli.mariaangela@unimore.it (M.A.V.); flavio.forni@unimore.it (F.F.); 3 Department of Biomedical, Metabolic and Neural Science, University of Modena and Reggio Emilia, 41124 Modena, Italy; eleonora.daini@unimore.it (E.D.); antonietta.vilella@unimore.it (A.V.); michele.zoli@unimore.it (M.Z.)

**Keywords:** PLGA, PEG, PF127, angiopep-2, nanoparticles, blood–brain barrier

## Abstract

The treatment of diseases that affect the central nervous system (CNS) represents a great research challenge due to the restriction imposed by the blood–brain barrier (BBB) to allow the passage of drugs into the brain. However, the use of modified nanomedicines engineered with different ligands that can be recognized by receptors expressed in the BBB offers a favorable alternative for this purpose. In this work, a BBB-penetrating peptide, angiopep-2 (Ang–2), was conjugated to poly(lactic-*co*-glycolic acid) (PLGA)-based nanoparticles through pre- and post-formulation strategies. Then, their ability to cross the BBB was qualitatively assessed on an animal model. Proof-of-concept studies with fluorescent and confocal microscopy studies highlighted that the brain-targeted PLGA nanoparticles were able to cross the BBB and accumulated in neuronal cells, thus showing a promising brain drug delivery system.

## 1. Introduction

Discovering new methods to treat diseases is becoming increasingly complicated because of the numerous natural biological barriers in our bodies such as opsonization by proteins in the blood, first-pass clearance organs, and the immune response [1,2]. The difficulty of overcoming these barriers to create a cure is exponentially increased when trying to deliver pharmaceutics across the blood–brain barrier (BBB) [3,4]. This is due to the brains increased security including tight junctions, low molecule diffusion rates, efflux transporters, and difficulty in reaching sufficient drug exposure in the brain compartment [5]. Nanomedicine has been the leading field in surpassing these barriers to deliver drugs to the brain. This is because their physico-chemical characteristics can be controlled to improve drug solubility, compatibility in BBB interactions, and can be targeted using ligands specific to improve delivery into the brain [6,7,8].

A very promising targeting ligand for the delivery of nanomedicines into the brain is Angiopep-2 (Ang–2), a 19 amino acid peptide (TFFYGGSRGKRNNFKTEEY-OH) which binds the low-density lipoprotein receptor-related protein 1 (LRP1), which is widely expressed throughout the central nervous system (CNS) (by endothelial cells on the basolateral surface, neuroblasts, microglia, astrocytes, and neurons) [9,10,11,12]. This ligand has been highly sought after in its use to deliver nanomedicines to the brain because of its ability to activate transcytosis across the BBB, as well as its upregulation in human glioma cells [13,14,15]. To this end, Ang–2 has been conjugated to numerous nanocarrier types in order to improve BBB crossing, including liposomes, tandem micelles, PEG-PCL, solid lipid nanoparticles, dendrigraft poly-l lysine, and poly-amidoamine dendrimers to deliver various active compounds (Coumarin, docetaxel, siRNA, etc.) [16,17,18,19].

Poly(lactic-*co*-glycolic acid) (PLGA) is an FDA approved polymer that can self-assemble into nanoparticles, with selectable features including the size, shape, charge, and drug loading capacity as a function of the formulation parameters. Moreover, literature extensively reported its ability to encapsulate, protect, and deliver a wide range of bioactive compounds (small molecules, proteins, enzymes, etc.) [20,21,22]. However, only a few studies indicated that PLGA-based nanoparticles modified with Ang–2 could be a promising active brain-targeting drug delivery system [13,14,18,23,24]. In this work, we focused on creating and characterizing targeted Ang–2 PLGA nanoparticles (NPs); the surface was engineered by conjugating Ang–2 directly to the polymer, and nanoformulation was then gained, or with post-formulation anchorage to NP through the conjugation of the thiol-containing cysteine added to the Ang–2 and a maleimide containing PLGA. These NPs were then characterized for their amount of Ang–2 bound to the NPs and tested in vivo for their ability to pass the BBB and enter into the brain parenchyma. Moreover, technological and methodological procedures to produce engineered nanomedicines proposed in our work are quite simple and easy to be adapted from brain delivery of other potential drugs, therefore spreading the possibility of application in other brain diseases.

## 2. Materials and Methods

### 2.1. Materials

Poly(lactic-*co*-glycolic acid) of molecular weight 24–38 kDa (PLGA 50:50) and Pluronic F127^®^ were purchased from Sigma-Aldrich (Milan, Italy). The PLGA-*b*-PEG copolymer was previously obtained through the modification of PLGA with mPEG-NH_2_ (MW: 5000 Da) [22]. The PLGA (Resomer 503 H) derivatized with the fluorophore Cy5 was obtained by the Laboratory of Nanomedicine and Pharmaceutical Technology of the University of Modena and Reggio Emilia (Modena, Italy) as previously reported [25]. The PLGA-Mal (MW: 20 kDa) was purchased from Nanosoft Biotechnology (Lewisville, TX, USA). The modified Angiopeptide-2 with a cysteine end group (CTFFYGGSRGKRNNFKTEEY), referred to from now as Ang–2, was synthesized by the company GenScript (MerckMillipore, Oregon City, OR, USA). The ultrapure water was supplied by a Milli-Q^®^ water purification system (Synergy^®^ UV, Darmstadt, Germany). All the solvents and other reactive products were also of a high degree of purity (>95%) and used as purchased without modification, unless otherwise stated.

### 2.2. Animals

For the in vivo tests, C57BL/6 mice were used. The experiments were carried out in accordance with the European Communities Council Directives of 24 November 1986 (86/609/EEC) for experimental care.

### 2.3. Synthesis and Characterization of PLGA Conjugate with the Ang–2

**Figure 1:**
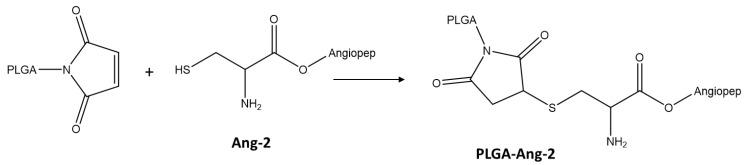
Scheme of PLGA functionalization with Ang–2.

The conjugation between Ang–2 and PLGA was carried out by reacting the maleimide group, present on the glycolic acid terminus of the PLGA chains, with the cysteine thiol group originating from the peptide, as shown in Figure 1 [4,26]. Briefly, 20 mg of PLGA-Mal together with 1.2 mg of the peptide (molar ratio PLGA-Mal/Ang–2 2:1) were dissolved in 4 mL anhydrous dimethyl sulfoxide (DMSO). After 15 h of reaction under constant agitation, the modified polymer was precipitated by adding diethyl ether [27]. Finally, the polymer was dried under reduced pressure and stored for characterization and use in the preparation of nanoparticles [28].

Modified PLGA products (PLGA-Ang–2) were analyzed by ^1^H-NMR to confirm the presence of Ang–2. The conjugate and control samples (3 mg) were dissolved in deuterated DMSO (0.5 μL), and the spectrum was obtained in a Bruker 400 MHz spectrometer.

### 2.4. Preparation of PLGA Nanoparticles Functionalized with Ang–2 (Ang–2-NPs)

**Figure 2:**
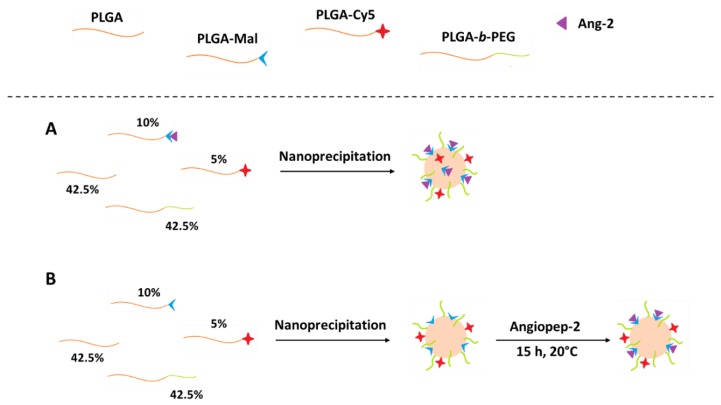
Schematic representation of the modification of PLGA/PLGA-*b*-PEG nanoparticles with Ang–2 through (**A**) pre-functionalization and (**B**) post-functionalization.

For comparative purposes, the polymer nanoparticles were obtained through the nanoprecipitation method using two different conjugation strategies: pre and post-functionalization (Figure 2) [29].

The pre-formulation functionalized nanoparticles (pre-formulation Ang–2 NPs) were prepared from mixtures with the following composition: PLGA (42.5%), PLGA-*b*-PEG (42.5%), PLGA-Cy5 (5%), and PLGA-Ang–2 (10%, synthesized as described above). An exact amount (10 mg) of the mixture was dissolved in 2 mL of acetone and subsequently added dropwise to 4 mL of a PF127 solution (1.0% *w*/*v*). Finally, the organic solvent was removed under reduced pressure using a rotary evaporator and purified as described in Section 2.5.

Post-formulation functionalized nanoparticles (post-formulation Ang–2 NPs) were prepared as previously described for pre-formulation Ang–2 NPs but using a modified polymer mixture: PLGA (45%), PLGA-*b*-PEG (45%), and PLGA-Mal (10%). After purification and resuspension, the nanoparticles obtained were subsequently modified through covalent binding with Ang–2 in different PLGA-Mal/Ang–2 molar ratios (3:1, 2:1, and 1:1). For this modification, the corresponding amount of Ang–2 was dissolved in an aqueous solution, added to the NPs suspension, and reacted under constant stirring for 15 h [7,29,30]. Finally, Cy5 fluorescently labeled nanoparticles were prepared with the following composition: PLGA (42.5%), PLGA-*b*-PEG (42.5%), PLGA-Cy5 (5%), and PLGA-Mal (10%), and they were post modified (PLGA-Mal/Ang–2 molar ratio 2:1) in order to carry out in vivo tests. Nanoparticles without PLGA-Mal were prepared as controls.

### 2.5. Purification of Nanoparticles

For removal and quantification of free Ang–2 from the post-formulation Ang–2 NPs, the suspensions obtained were centrifuged for 15 min at a speed of 13,500 rpm using a Spectrafuge™ 24D microcentrifuge (LabNet, Woodbridge, NJ, USA). The supernatant was removed by decantation, and the solid was resuspended in Milli-Q water (MerckMillipore, Oregon City, OR, USA). The Ang–2-NP suspension was then stored in the refrigerator for further analysis.

### 2.6. Characterization of Nanoparticles

#### 2.6.1. Distribution of Particle Size and Zeta Potential

Particle size, polydispersity index and zeta potential measurements were carried out on a Zetasizer Nano ZS (Malvern Instrument, Worcestershire, UK) at 25 °C, after purification of the NPs. The experiments were performed in triplicate, and the results are reported as the mean value ± standard deviation.

#### 2.6.2. HPLC Quantification of Ang–2 in Post-Functionalized Nanoparticles

The quantification of Ang–2 conjugated after NP formation (post-formulation) was carried out indirectly by quantifying the amount of free Ang–2 that remained in the supernatant after NP purification using high-performance liquid chromatography (HPLC). A 500 μL aliquot of supernatant was left at RT for 72 h until complete dimerization was achieved, and 50 μL was injected onto a C8 column (Aeris™ WIDEPORE XB-C8, 150 × 4.6 mm, 3.6 μm) using a gradient method where the mobile phases consisted of 0.1% trifluoroacetic acid (TFA) in water (solution A) and 0.1% TFA in acetonitrile (ACN) (solution B), where solution B was increased from 10% to 35% over 12 min with a flow of 1.2 mL/min. The amount of Ang–2 in the supernatant was determined with a standard curve designed by integrating the peak of interest using a UV detector monitoring λ = 215 nm (linear in the range of 1–50 μg/mL and a correlation coefficient of 0.9991). The difference between the initial amount of peptide added to the formulation and the amount quantified in the supernatant allowed the calculation of the percentage of peptide bound to the NPs.

### 2.7. In Vivo Tests: Brain Uptake of the Nanoparticles

#### 2.7.1. Animal Handling Protocols and Sample Preparation for Systemic Injection of Post-Functionalized Ang–2 NPs

The post-formulation procedure lead to the production of post-formulation Ang–2 NPs (or as a control, fresh saline solution lacing NPs), which were suspended in a saline solution and injected i.p. (100 μL, 1 mg/mL) into C57Bl6 mice (female and male, weight close to 25 g). As control, unmodified NPs were also administered. After 1 or 4 h, the mice were sacrificed for histological processing: each mouse was anesthetized with chloral hydrate (400 mg/kg, i.p.) and underwent intracardial perfusion with an infusion of 50 mL of 0.9% NaCl saline containing heparin sodium (5000 U/L) followed with 4% paraformaldehyde and 0.2% picric acid in phosphate-buffered saline (PBS) (70 mL/7 min) ensued by extraction of the brain. The brains were post-fixed in the same solution for 12 h, rinsed with increasing concentrations of sucrose in PBS over 1.5 d, and frozen using dry ice. Coronal sections (50 μm thick) were cut at a cryotome, washed in cold 1× PBS, and stored at −20 °C in a glycerol-PBS solution until used.

#### 2.7.2. Immunohistochemistry of Brain Sections

Brain sections were processed for multiple immunofluorescence histochemistry according to published protocol (ref). Neuronal nuclear antigen (NeuN) (Millipore, USA) from DAKO was used as the primary antibody, and goat anti-mouse Alexa488 (1:200) or goat anti-rabbit Alexa488 (1:200) were used as secondary antibodies.

#### 2.7.3. Confocal Analysis

Confocal analysis was performed with a Leica DM IRE 2 (Bannockburn, IL, USA); Leica Confocal System: scan head multiband 3 channels Leica TCS SP2 with Acousto-Optical Beam Splitter (AOBS) laser diode blu (405 nm/25 mW), Laser Ar (458 nm/5 mW) (476 nm/5 mW) (488 nm/20 mW) (496 nm/5 mW) (514 nm/20 mW), Laser HeNe (543 nm/1.2 mW), Laser HeNe (594 nm) (orange), and Laser HeNe (633 nm/102 mW). In particular, NPs (due to their labeling with Cy5) were clearly visible by excitation with the 633 nm laser with an emission readout at 650 nm, while staining of NeuN and other immunohistochemistry tags were detected using 488 and 500 nm excitation and emission wavelengths, respectively.

## 3. Results and Discussion

### 3.1. Quantification of Ang–2 on Modified PLGA by ^1^H-NMR

**Figure 3:**
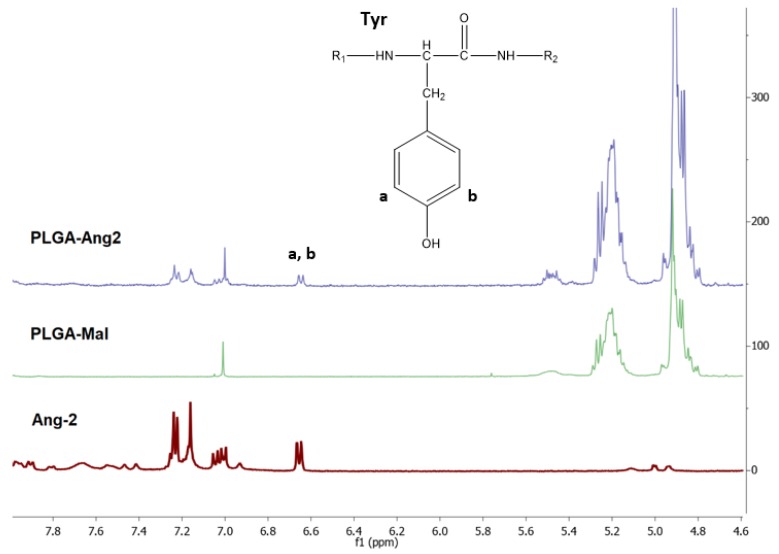
^1^H-NMR spectra of PLGA-Mal, Ang–2, and PLGA-Ang2 obtained in deuterated DMSO.

The ^1^H-NMR spectra of PLGA-Mal, Ang–2, and the PLGA modified with Ang–2 (PLGA-Ang–2) are presented in Figure 3. The intense signals observed at δ = 4.9 and 5.2 ppm in the PLGA-Mal and PLGA-Ang–2 spectra correspond to the methylene (–CH_2_) and methine (–CH) groups of the PLGA. On the other hand, the signals observed in the PLGA-Ang–2 spectrum around δ = 7.0 ppm are attributed to the aromatic protons present in the amino acids phenylalanine (Phe) and tyrosine (Tyr) of the peptide, thus confirming the polymer functionalization [28].

The functionalization degree of PLGA with Ang–2 was calculated from the ratio between the area of the doublet observed at δ = 6.65, corresponding to aromatic protons of Tyr, and the area of the signal observed at δ = 1.5, corresponding to the protons of the methyl groups (–CH_3_) of PLGA. In Equation (1), the obtained Ang–2/PLGA relation is shown, where *a_i_* corresponds to the integrated area under the signals of the ^1^H-NMR spectrum for the respective fractions, *m_i_* corresponds to the number of protons corresponding to each signal, and *n_i_* is the number of repetition units of the fraction i. This calculation indicates that for every PLGA there is 0.51 units of Ang–2 (2:1 PLGA/Ang–2). The modified polymer was used to prepare the pre-functionalized nanoparticles.

(1) $$\frac{Ang–2}{PLGA}=\frac{a_{Ang–2\;}/m_{Ang–2\;}}{\;(a_{Ang–2\;}/m_{Ang–2\;})+(a_{CH_{3}\;}/(m_{CH_{3}\;}\times \;n_{CH_{3}\;}))}=\frac{4/4}{\;\left(4/4\right)\;+\;(795.29/\left(3\times 278\right))}=0.51$$

### 3.2. HPLC Quantification of Ang–2 in Post-Functionalized Nanoparticles

**Figure 4:**
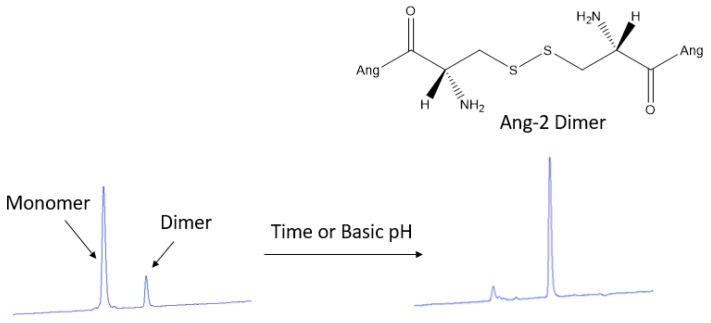
Chromatographic analysis obtained for the monomer (2.4 min) and the Ang–2 dimer (3.4 min) after 72 h at room temperature or adding a tris buffer solution at pH 9 only to quickly check the dimer formation.

Because of the incompatibility of PLGA with HPLC analysis, the amount of Ang–2 bound to the post-functionalized nanoparticles was determined indirectly from the supernatant obtained during the purification process using a calibration curve previously constructed from a standard solution of the peptide. In the chromatograms obtained for the supernatant, two peaks were detected at 2.7 and 3.4 min of retention. By analyzing this solution over time, it is possible to observe the time-dependent transformation of the first eluted peak into the second, as shown in Figure 4. This conversion of the product can be explained by disulfide bond formation (dimerization) of the Ang–2 peptide N-terminal cysteines [31]. Since the formation of the Ang–2 dimer is spontaneous, the complete conversion of the monomer to the dimer was allowed, and the dimer calibration curve was constructed to carry out the quantification of the free Ang–2.

Table 1 shows the amount of Ang–2 bound to the nanoparticles for the different ratios of PLGA-Mal/Ang–2 studied (3:1, 2:1, and 1:1). It was observed that even when PLGA-Mal was not used in the formulation (no reaction should occur between Ang–2 and PLGA), part of the Ang–2 remained bound to the nanoparticles. This is due to the nonspecific adsorption of the peptide to the NP surface. On the other hand, the amount of Ang–2 bound to the NPs increased significantly (*P* < 0.05) with the presence of PLGA-Mal in the formulation, which suggests the covalent binding of this peptide with the polymer. Finally, and as expected, an increase in the amount of Ang–2 per grams of NP was observed by increasing the amount of initial peptide added to the formulation.

### 3.3. Size Distribution and Zeta Potential of Pre- and Post-Functionalized Ang2-NPs

The pre- and post-functionalized Ang2-NPs were characterized through particle size, polydispersity index, and zeta potential measurements. For comparative purposes, the values of these variables for NPs without Ang–2 were also determined (Table 2). For all prepared NPs, monomodal and homogeneous dispersions were obtained (PDI ≤ 0.1); however, it was observed that the conjugation of Ang–2 to the nanoparticles through either strategy led to an increase in particle size compared to non-functionalized nanoparticles (*P* < 0.05), which could be related to the presence of peptide onto NPs. The zeta potential values were lower than −20 mV for all formulations because of the negative surface charge related with the carboxylic groups of the PLGA.

### 3.4. In Vivo Brain Distribution of ANG-2 NPs

**Figure 5:**
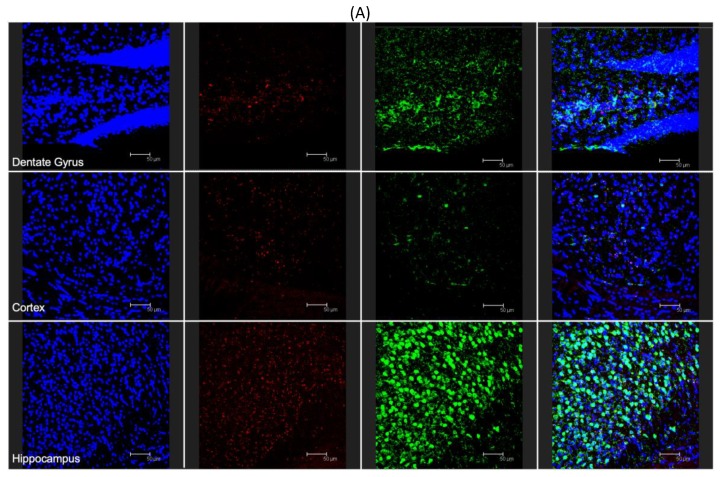

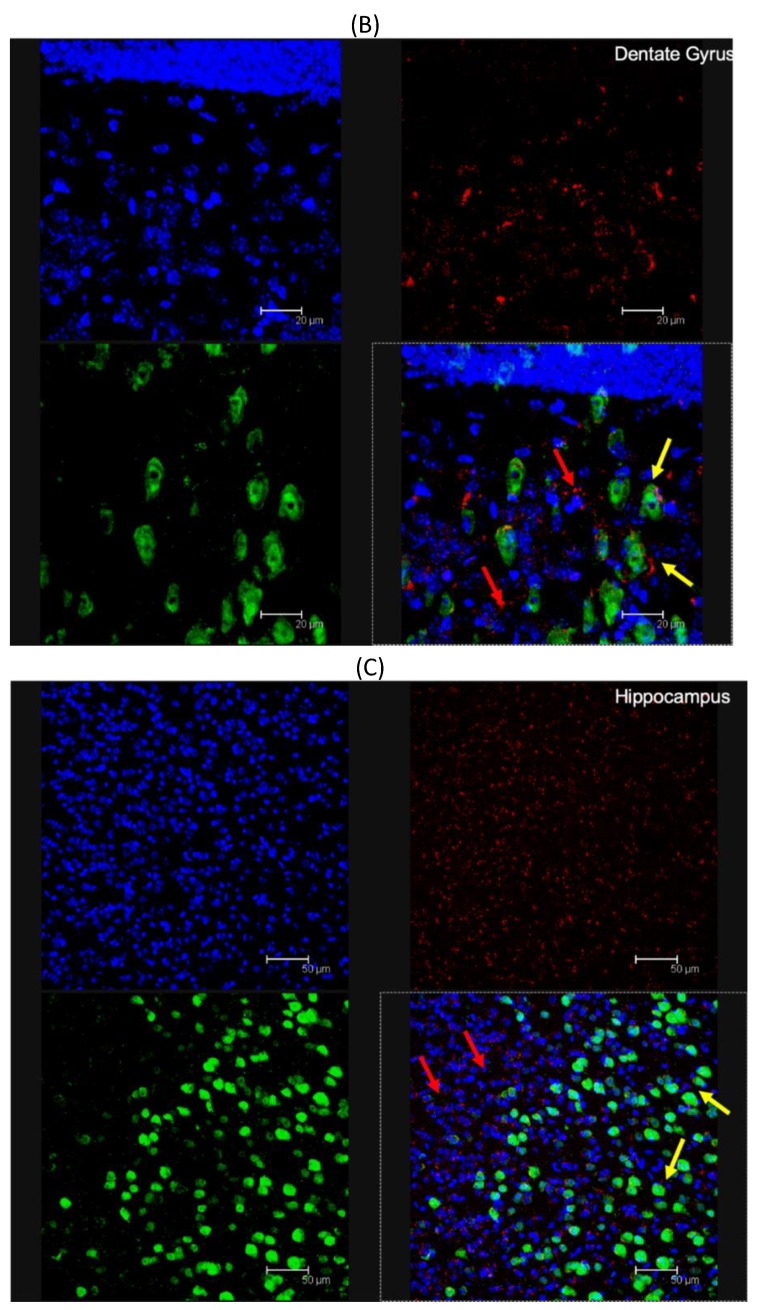
(**A**) Fluorescent microscopy analysis of Ang–2-NP brain distribution: cross section of the dentate gyrus, cortex, and hippocampus stained with DAPI (blue channel), Cy5- labeled ang-2-NPs (red channel), and NEUN (green channel). (**B**) Magnified analysis of the dentate gyrus. (**C**) Magnified analysis of hippocampus. In both images (**B**,**C**), colocalization with (red arrows) NEUN negative stained cells and colocalization with neurons (yellow arrows) are identified. All images are representative of the average analysis, and scale bars in (**A**–**C**) are set at 50, 20, and 50 µm, respectively.

To demonstrate the ability for modified nanoparticles to cross the blood–brain barrier and reach various brain areas, 100 μL of post-formulation Ang–2 NP suspension (1 mg/mL) was i.p. injected into C57BL/6 mice. After one or four hours, the mice were sacrificed and the brain was removed and histologically stained for the presence of cell nuclei (DAPI) and neuronal cells (neuronal nuclear antigen, NeuN). Representative results were already visible, qualitatively demonstrating penetration of the Ang–2-NPs across the BBB at 1 h (data not shown), similar to those results obtained at 4 h. With respect to control samples (unmodified labeled NPs) not showing significant signals related to NPs (Figure S1), the presence of Ang–2 NPs was uniform throughout the dentate gyrus, cortex, and hippocampus (Figure 5A, red channel), suggesting a robust, uniform passage of the NPs across the BBB. The clear accumulation of Ang–2 NPs in brain parenchyma is remarkable in consideration of the inability of unmodified NPs and modified NPs used as controls (data not shown) to cross BBB alone (data not shown), which was also broadly assessed from other outputs in literature of NPs of similar composition and size [30,32]. In analyzing the images, Ang–2-NPs colocalized with the various cell types present in the brain, evidenced only with DAPI, but were often also in close proximity to the neuronal cells (Figure 5B, red and yellow arrows respectively). This is interesting because it could indicate a different mode of cell uptake than what has been seen previously for PLGA NPs targeted with the simil-opioid peptide ligand g7 [20,27,33] that are broadly up-taken only by neurons. Moreover, further studies will be required to better elucidate the mechanism of Ang–2 NP entrance in the brain or in the cells, for instance by blocking endo-transcytosis or the clathrin/caveolin uptake process, and therefore to draft a complete hypothesis on BBB-crossing pathways and neuron uptake of these kinds of NPs. Transcytosis pathways, therefore, could not be evidenced with these remarkable but preliminary experiments.

## 4. Conclusions

In summary, in this work, Ang–2 NPs were prepared by nanoprecipitation using pre- and post-formulation strategies and using PF127 as a stabilizer. With both methodologies, we obtained NPs with sizes lower than 200 nm, compatible with systemic administration and to enable possible BBB crossing. Furthermore, brain accumulation was, for the first time, confirmed through in vivo analysis, where we observed the localization of this kind of nanoparticle within the brain cells, highlighting in particular the neuron accumulation in different brain areas (i.e., cortex and hippocampus). In fact, even if Ang–2 is not new in the CNS targeting panorama, previous papers dealt with experiments on different polymers used in production of nanomedicines (as caprolactone [16], a mixture of mesoporous silica PLGA-based NPs [34]), or different nanocarrier architectures, as in the use of micelles [35]. In any paper, besides some in vivo experiments on BBB crossing, data on cell tropism and clear brain visualization are not often reported. Therefore, the formulations presented in the current paper could be used as carriers of different drugs to the CNS, thus increasing the alternatives for the treatment of brain diseases.

## Figures and Tables

**Table 1:** Amount of Ang–2 bound to the nanoparticles determined through HPLC for three different PLGA-Mal/Ang–2 ratios. The controls correspond to nanoparticles with the same amount of Ang–2 as the experiment, but without the presence of PLGA-Mal. Values represent mean ± standard deviation (*n* = 3 experiments). * and ** show statistically significant differences (*P* < 0.05) between the label samples.

Initial Amount PLGA-Mal/Ang–2	µg Ang–2/g NPs	Final Molar Ratio Ang–2/PLGA-Mal
Control (3:1)	1.65 ± 0.60	
3:1	3.06 ± 0.11	0.25 ± 0.01
Control (2:1)	2.59 ± 0.71 *	
2:1	4.42 ± 0.74 *	0.37 ± 0.06
Control (1:1)	4.24 ± 0.71 **	
1:1	8.78 ± 1.93 **	0.73 ± 0.23

**Table 2:** Particle size, polydispersity index, and zeta potential of PLGA-*b*-PEG nanoparticles pre- and post-functionalized with Ang–2.

PLGA-*b*-PEG Formulations	Particle Size (nm)	PDI	Zeta Potential (mV)
Non-functionalized NPs	136.3 ± 6.8	0.06 ± 0.01	−27.4 ± 2.7
Pre-Formulation Ang–2 NPs	166.4 ± 2.4	0.08 ± 0.04	−26.2 ± 0.9
Post-Formulation Ang–2 NPs	177.3 ± 12.7	0.10 ± 0.01	−21.9 ± 3.4

## Supplementary Materials

The following are available online at https://www.mdpi.com/1999-4923/12/1/72/s1, Figure S1: Fluorescent microscopy analysis of non-functionalized NP brain distribution. Staining with DAPI (blue channel), Cy5- fluorescence (red channel), and NEUN (green channel).
