# Supplementary material for: PLGA-PEG-ANG-2 Nanoparticles for Blood–Brain Barrier Crossing: Proof-of-Concept Study
Source: Pharmaceutics. 2020 Jan 17;12(1):72. doi: 10.3390/pharmaceutics12010072 (PMC7023215; doi:10.3390/pharmaceutics12010072)
Supplement: Supplementary file 1 [file pharmaceutics-12-00072-s001.zip › pharmaceutics-668447-supplementary.pdf]

pharmaceutics

MDPI

Supplementary Materials: PLGA-PEG-ANG-2 Nanoparticles for Blood–Brain Barrier Crossing: Proof-of-Concept Study

Gina P. Hoyos-Ceballos, Barbara Ruozi, Ilaria Ottonelli, Federica Da Ros, Maria Angela Vandelli, Flavio Forni, Eleonora Daini, Antonietta Vilella, Michele Zoli, Giovanni Tosi, Jason T. Duskey and Betty L. López-Osorio

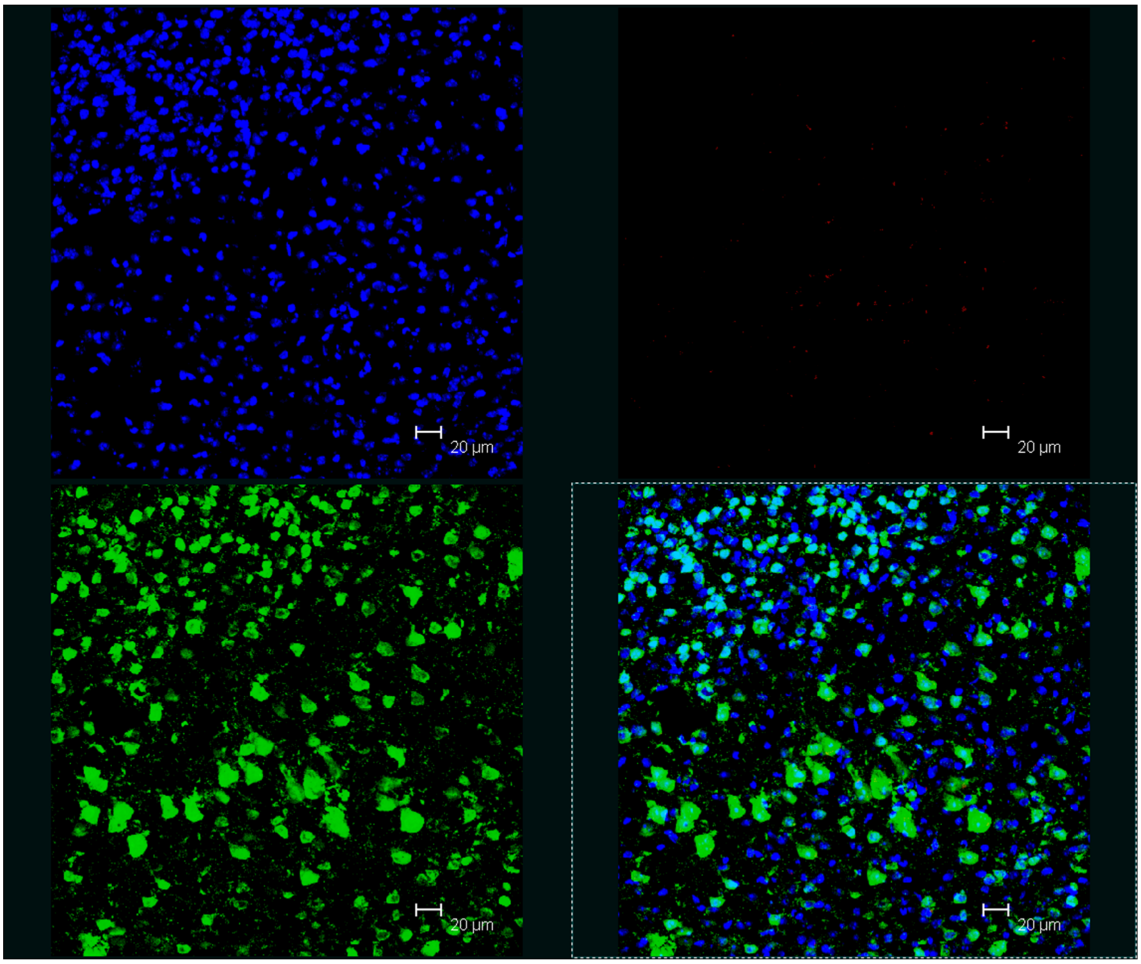

Figure S1. Fluorescent microscopy analysis of non-functionalized NP brain distribution. Staining with DAPI (blue channel), Cy5 fluorescence (red channel), and NEUN (green channel).
